# PTBP1 acts as a dominant repressor of the aberrant tissue‐specific splicing of *ISCU* in hereditary myopathy with lactic acidosis

**DOI:** 10.1002/mgg3.413

**Published:** 2018-09-12

**Authors:** Denise F. R. Rawcliffe, Lennart Österman, Angelica Nordin, Monica Holmberg

**Affiliations:** ^1^ Unit for Medical and Clinical Genetics Department of Medical Biosciences Umeå University Umeå Sweden

**Keywords:** alternative splicing, hereditary myopathy, *ISCU*, PTBP1

## Abstract

**Background:**

Hereditary myopathy with lactic acidosis (HML) is an autosomal recessive disease caused by an intron mutation in the iron‐sulfur cluster assembly (*ISCU*) gene. The mutation results in aberrant splicing, where part of the intron is retained in the final mRNA transcript, giving rise to a truncated nonfunctional ISCU protein. Using an *ISCU* mini‐gene system, we have previously shown that PTBP1 can act as a repressor of the mis‐splicing of *ISCU*, where overexpression of PTBP1 resulted in a decrease of the incorrect splicing. In this study, we wanted to, in more detail, analyze the role of PTBP1 in the regulation of endogenous *ISCU* mis‐splicing.

**Methods:**

Overexpression and knockdown of PTBP1 was performed in myoblasts from two HML patients and a healthy control. Quantification of *ISCU* mis‐splicing was done by qRTPCR. Biotinylated *ISCU* RNA, representing wildtype and mutant intron sequence, was used in a pull‐down assay with nuclear extracts from myoblasts. Levels of PTBP1 in human cell lines and mice tissues were analyzed by qRTPCR and western blot.

**Results:**

PTBP1 overexpression in HML patient myoblasts resulted in a substantial decrease of *ISCU* mis‐splicing while knockdown of PTBP1 resulted in a drastic increase. The effect could be observed in both patient and control myoblasts. We could also show that PTBP1 interacts with both the mutant and wild‐type *ISCU* intron sequence, but with a higher affinity to the mutant sequence. Furthermore, low levels of PTBP1 among examined mouse tissues correlated with high levels of incorrect splicing of *ISCU*.

**Conclusion:**

Our results show that PTBP1 acts as a dominant repressor of *ISCU* mis‐splicing. We also show an inverse correlation between the levels of PTBP1 and *ISCU* mis‐splicing, suggesting that the high level of mis‐splicing in the skeletal muscle is primarily due to the low levels of PTBP1.

## 1 INTRODUCTION

Hereditary myopathy with lactic acidosis (HML, OMIM#255125) is an autosomal recessive disease characterized by a low tolerance to exercise from an early age (Larsson, Linderholm, Müller, Ringqvist, & Sörnäs, 1964; Mochel et al., 2008; Olsson, Lind, Thornell, & Holmberg, 2008). Low‐level exercise can trigger an array of symptoms, such as muscle cramps, palpitations, and dyspnea, as well as an increased release of pyruvate and lactate (Drugge, Holmberg, Holmgren, Almay, & Linderholm, 1995; Larsson et al., 1964). At the biochemical level, the patients display decreased levels and activity of complex I, II, and III of the electron transport chain (ETC), as well as mitochondrial aconitase (Hall, Henriksson, Lewis, Haller, & Kennaway, 1993; Haller et al., 1991; Mochel et al., 2008; Nordin, Larsson, Thornell, & Holmberg, 2011; Sanaker et al., 2010). Extreme exercise in combination with strict diets can trigger severe episodes of disease characterized by rhabdomyolysis, myoglobinuria, as well as life‐threatening levels of lactic acid (Kollberg, Melberg, Holme, & Oldfors, 2011; Larsson et al., 1964).

The disease is caused by an intronic G>C mutation (NC_000012.12 (NM_213595):c.418 + 382G>C) in the iron‐sulfur cluster assembly gene (*ISCU*, OMIM *611911) (Kollberg et al., 2009; Mochel et al., 2008; Olsson et al., 2008). The mutation is located 382 bp downstream of exon 4 and activates a cryptic splice site within the intron, resulting in the inclusion of an 86 or 100 bp long pseudoexon in the final mRNA transcript (Kollberg et al., 2009; Mochel et al., 2008; Olsson et al., 2008; Sanaker et al., 2010). The incorrectly spliced mRNA is translated into a truncated nonfunctioning ISCU protein with 15 novel amino acids followed by a premature stop, which interrupts the structure of the last α‐helix (Kollberg et al., 2009; Mochel et al., 2008; Olsson et al., 2008). The ISCU protein functions as a scaffolding protein in the formation of iron–sulfur (Fe–S) clusters (Agar et al., 2000; Lill et al., 2012; Rouault & Maio, 2017; Rouault & Tong, 2008). Fe–S clusters are essential cofactors with electron transferring abilities and can be found in a wide range of proteins including complex I, II, and III of the ETC, as well as in mitochondrial aconitase in the TCA cycle (Agar et al., 2000; Crooks et al., 2012). Even though the *ISCU* mutation affects the performance of the ETC, the HML patients show a muscle‐specific phenotype, leaving other energy‐demanding organs, such as the heart and the central nervous system, unaffected (Kollberg et al., 2011; Larsson et al., 1964). ISCU protein is almost absent in muscle tissue from HML patient, but not in other tissues examined (Kollberg et al., 2009; Mochel et al., 2008; Nordin et al., 2011). Lower levels of ISCU protein has also been observed in patient myoblast and fibroblasts, but the decrease is less pronounced in fibroblasts (Crooks et al., 2012; Sanaker et al., 2010).

Besides the mutation identified in HML, two other *ISCU* mutations that cause myopathies have been identified. Two brothers of Swedish/Finish decent have been shown to be compound heterozygous for the HML intronic mutation and a recessive missense mutation, c.149G>A (Kollberg et al., 2009). Recently, a de novo *ISCU* missense mutation, c.287G>T, that resulted in a dominant form of myopathy, was reported in a 23‐year‐old Italian male (Legati et al., 2017). In both cases, the mutations resulted in a more severe and progressive phenotype with additional symptoms aside from the myopathy (Kollberg et al., 2009; Legati et al., 2017). The clinical phenotype observed for the two missense mutations still suggests that skeletal muscle in general is more sensitive to ISCU deficiency than many other tissues. In the case of HML, the muscle‐specific phenotype can be explained by the fact that the highest level of incorrect splicing, around 80%, is found in skeletal muscle (Crooks et al., 2012; Nordin et al., 2011). In contrast, only 30% and 10% mis‐splicing is observed in the heart and liver, respectively (Nordin et al., 2011). Furthermore, transgenic mice expressing human *ISCU*, show the highest levels of incorrect splicing in the slow fiber soleus muscle and serine and arginine‐rich splicing factor 3 (SRSF3, OMIM*603364) has been suggested to be involved in this muscle‐type‐specific splicing (Rawcliffe, Osterman, Lindsten, & Holmberg, 2016).

Polypyrimidine tract‐binding protein 1 (PTBP1, OMIM*600693) has previously been suggested to act as a repressor of *ISCU* mis‐splicing. Using an *ISCU* mini‐gene system, overexpression of *PTBP1* was shown to markedly decrease the mis‐splicing (Nordin, Larsson, & Holmberg, 2012). In this study, we investigated the regulatory role of PTBP1 in *ISCU* mis‐splicing in more detail. Levels of *Ptbp1* in different tissues were correlated to the level of mis‐splicing and we could show a negative correlation between *ISCU* mis‐splicing and *Ptbp1* levels. Furthermore, using myoblasts from HML patients, we analyzed the effect of overexpression and knockdown of *PTBP1* on the mis‐splicing of *ISCU* in myoblasts from HML patients. Overexpression of *PTBP1* drastically decreased the levels of incorrectly spliced *ISCU*, while knockdown of *PTBP1* increased the incorrect splicing. Taken together, our results indicate that PTBP1 is a major determinant in the pathology of HML, where the levels of PTBP1 in a tissue controls the levels of mis‐splicing of mutant *ISCU*.

## 2 MATERIALS AND METHODS

### 2.1 Mice

*ISCU* transgenic mice (Rawcliffe et al., 2016) from a CBA/B6 background were kept in standard cages with free access to water and food (CRM Expanded, SDS). Mice of both genders were sacrificed by cervical dislocation at 9 weeks of age, and the organs of interest were collected and immediately frozen in N_2_ (l) followed by storage in −80°C. All procedures were approved by the Ethical Committee for Animal Research at Umeå University (A5‐12, A74‐14).

### 2.2 Cells

Primary myoblasts were isolated from the tibialis anterior muscle of two HML patients (P1, P2) and a healthy control (C1). The myoblasts were cultured in 4 volumes of Dulbecco's modified essential medium (DMEM, Gibco, Waltham, MA, USA) to 1 volume of Medium 199 (Gibco) supplemented with 20% FBS (Gibco), 5 ng/ml recombinant human hepatocyte growth factor (Invitrogen, Waltham, MA, USA) and 50 *μ*g/ml gentamycin (Sigma‐Aldrich, St. Louis, MO, USA). RD4, HeLa and HEK293T Lenti‐X cells (Clontech, Mountain View, CA, USA) were cultured in DMEM (Gibco) supplemented with 1% Glutamax (Gibco), 10% FBS, and 1% PenStrep (Gibco). All cells were cultured at 37°C with 5% CO2. Studies including human cells were approved by the Regional Ethics Committee for Medical Research at Umeå University (09‐105M). Written informed consent was obtained from all participants.

### 2.3 RNA isolation

RNA was prepared from 30 mg frozen mouse tissue in RNA lysis buffer with a stainless‐steel bead (5‐mm diameter) using TissueLyzer LT (Qiagen, Valencia, CA, USA) according to the manufacturer's instructions (RNeasy Fibrous Tissue Mini Kit; Qiagen). In brief, the samples were then digested with proteinase K followed by RNA isolation using the RNeasy Mini Spin columns (Qiagen). RNA was eluted in RNase‐free water and stored at −80°C. The NucleoSpin RNA plus kit was used to prepare RNA from cells according to manufacturer's instructions (Machery‐Nagel, Düren, Germany). In brief, lysis buffer was added directly to the myoblasts and collected followed by the removal of the genomic DNA by using a gDNA removal column. RNA was then isolated using an RNA plus column, eluted in RNase‐free water and stored at −80°C.

### 2.4 cDNA synthesis

cDNA was synthesized using the SuperScript III RT First‐Strand Synthesis System according to manufacturer's instructions (Invitrogen). In brief, approximately 1,200 ng of RNA from the mouse tissue or 275 ng of RNA from the myoblasts was incubated at 65°C for 5 min with 1 mmol/L dNTP and 50–100 ng random hexamers before the addition of 50 units of SuperScript III RT enzyme with 10 mmol/L DTT, 5 mmol/L MgCl_2_ in the supplied buffer. Samples were then incubated for 5 min at 25°C and for 1 hr at 50°C, followed by 15 min at 70°C to stop the reaction. The cDNA was stored at −20°C.

### 2.5 qRTPCR

cDNA was amplified by qPCR using SYBR green (Roche, Basel, Switzerland) with a CFX Connect Real‐Time PCR Detection System (Bio‐Rad, Hercules, CA, USA). Primers used to amplify human genes *PTBP1*(NC_000019.10), *SRSF3* (NC_000006.12), *ACTB* (NC_000007.14), and *ISCU* (NC_000012.12) were: PTBP1F (5′– TCCCAGATATAGCCGTTGGT –3′), PTBP1R (5′– CTTGCTGTCATTTCCGTTTG –3′), SRSF3F (5′– AGAGCTAGATGGAAGAACACT –3′), SRSF3R (5′– ATAATCATCTCGAGGGCGAC –3′), ACTBF (5′– GCACAGAGCCTCGCCTT –3′), ACTBR (5′– CCTTGCACATGCCGGAG –3′), ISCUExon3F (5′– ATGAAAAGGGGAAGATTGTGG –3′), ISCU_corrR (5′– GCATCTTCAGCCAGCATGGA –3′), and ISCU_incorrR (5′– TGGAAACAGCACAGATTTGGA –3′). Primers used to amplify the mouse genes *Ptbp1* (NC_000076.6), *Ptbp2* (NC_000069.6), and *Gapdh* (NC_000072.6) were: mPtbp1F (5′– GTCCCAGACATAGCAGTCGG –3′), mPTBP1R (5′– GCTCCTGTTGTCACCTTTGA –3′), mPtbp2F (5′– GAGGTTGCTGTTGGTGTGAA –3′), mPtbp2R (5′– GCCCCATCCATTTTATCTTCT –3′), mGapdhF (5′– TGCCCCCATGTTTGTGATG –3′), and mGapdhR (5′– TGTGGTCATGAGCCCTTCC –3′). An average cycle threshold (Ct) was calculated for each triplicate sample, which was transformed to 2^(−Ct)^ for all further calculations. Primer pair efficiencies were obtained from the standard curves based on 4‐point, 10‐fold dilutions of either pooled myoblast or mouse cDNA in a representative Ct range. Gene expression was calculated using 2^(−ddCt)^, where β‐actin for the human samples and mouse *Gapdh* for mouse samples was used as a reference gene. Splicing percentages of *ISCU* were calculated by taking the 2^(−dCt)^ value representing the incorrect splicing divided by the sum of the 2^(−dCt)^ values for the incorrect and correct splicing. The reverse primers ISCU_corrR and ISCU_incorrR targets the cDNA representing correct and incorrect *ISCU* mRNA, respectively, and are paired with ISCUExon3F for amplification of endogenous *ISCU*. Data were analyzed using Bio‐Rad CFX Manager software Version 3.1 (Bio‐Rad). All primers were purchased from Sigma‐Aldrich.

### 2.6 Cloning of PTBP1 expression vector and shPTBP1 vector

The *PTBP1* expression vector used in the overexpression assay was constructed by cloning the coding sequence for *PTBP1* into the pLM backbone of the pLM‐fSV2A vector (#27512; Addgene, Cambridge, MA, USA). For knockdown of *PTBP1*, a shPTBP1 DNA hairpin with a target sequence of GCGTGAAGATCCTGTTCAATA was cloned into the pLKO.1 plasmid (#10878; Addgene) according to manufacturer's instructions.

### 2.7 Lentiviral transduction of myoblasts

HEK293T Lenti‐X cells (Clontech, Mountain View, CA, USA) were transfected by calcium phosphate precipitates as described previously by Rawcliffe et al. (2016). In brief, a total of 55 *μ*g of lentiviral DNA vectors (Addgene), such as psPAX2 (virus envelope), pMD2.G (virus proteins) and DNA of interest, were used in a ratio of 1:0.7:1 in a total volume of 1,500 *μ*l with 250 mmol/L CaCl_2_. 2X HEPES (pH 7.00) of the same volume was added dropwise to the DNA mixture while air was bubbled through the solution. The mixture was left in RT for 30 min and then added dropwise to the HEK293T cells. After repeated media changes and addition of sodium butyrate, virus was collected 48‐hr posttransfection, frozen in N2 (l) and stored at −80°C.

Prior to all infections, the myoblasts were given fresh media with 10 *μ*g/ml polybrene (Millipore) followed by 180 *μ*l concentrated virus per well of a 6‐well plate where each well contained 80–100,000 myoblasts. For overexpression experiments, the cells were harvested for RNA and protein 48 hr after transduction. Virus with mCherry DNA was used as a control, as well as to assess the infection efficiency. For knockdown experiments, the myoblasts were given fresh media with 1.8 *μ*g/ml puromycin 24 hr after the infection. Virus with a scrambled shRNA (#1864; Addgene) was used as a control. The RNA and protein were harvested from the cells 72 hr after transduction. All chemicals were purchased from Sigma‐Aldrich unless otherwise stated.

### 2.8 Nuclear extracts

Frozen mouse tissue (50 mg) was disrupted using a Dounce homogenizer in Buffer A (10 mmol/L HEPES pH 7.9, 1.5 mmol/L MgCl_2_, 10 mmol/L KCl) with freshly added 1 mmol/L DTT and a protease inhibitor cocktail (Complete, Roche, Basel, Switzerland). Myoblasts or RD4 cells were pelleted at 300×g for 10 min at 4°C, washed with cold PBS and pelleted again. The cell pellet was resuspended in Buffer A and incubated 15 min on ice. Cell samples were then vortexed in pulses for 10 min while homogenized tissue samples were vortexed for 10 s in 0.5% NP‐40. Nuclei were pelleted at 4,500×g for 20 s and washed with Buffer C (20 mmol/L HEPES pH 7.9, 1.5 mmol/L MgCl_2_, 420 mmol/L NaCl, 0.2 mmol/L EDTA, and 10% glycerol) including protease inhibitors (Complete, Roche) and phosphatase inhibitors (10 mmol/L NaF, 10 mmol/L β‐glycerophosphate, 1 mmol/L sodium vanadate). Pellets were resuspended in Buffer C and vigorously shaken on ice for 30 min followed by centrifugation at 14,000×g for 10 min at 4°C. The supernatant, which is the nuclear extract fraction, was collected. Protein concentrations were determined by standard BCA assays (Pierce, Waltham, MA, USA). For the western blot assay, 400 *μ*g of nuclear extract from mouse tissues was loaded into each well. All chemicals were purchased from Sigma‐Aldrich unless otherwise stated.

### 2.9 MACS biotinylated molecule isolation

The μMACS Streptavidin kit (MACS Miltenyi Biotec, Bergisch Gladbach, Germany) was used to capture RNA‐binding factors as described by Rawcliffe et al., 2016. *ISCU* oligos representing normal and mutated *ISCU* were used, where the mutated sequence has a C instead of a G, (5′–[Biotin]AGCUCCAAUCUUU**C**/**G**AUUUCAGAAUCUG–3′). A scrambled RNA oligo was used as a negative control (5′– [Biotin]AUCGUGGAUAUAGCAGCGUACUAGUAG–3′). In brief, the binding reaction, including myoblast nuclear extract, biotinylated RNA oligos, and streptavidin microbeads (MACS Miltenyi Biotec), was added to μMACS columns attached to a magnetic MACS multi‐stand (MACS Miltenyi Biotec). The column was washed and the bead‐captured nuclear factors were eluted with 150 *μ*l PBS. For the western blot assay, the maximum volume of 33 *μ*l of eluent was loaded into each well, while 60 *μ*g of myoblast nuclear extract and 15 *μ*g of RD4 nuclear extract were used as positive controls. All chemicals were purchased from Sigma‐Aldrich unless otherwise stated.

### 2.10 Western blot analysis

Cells were lyzed in protein lysis buffer (2% SDS, 100 mmol/L Tris‐HCl, pH 6.8) and protein concentration was determined by a BCA assay (Pierce). Samples (60 *μ*g) were denatured in sample buffer and were size separated using Bis‐Tris precast 4%–12% gels (Bio‐Rad, Hercules, CA, USA). The proteins were blotted onto an Amersham Hybond‐ECL membrane (GE Healthcare, Fairfield, CT, USA) for 45 min at 15 V. Membranes were blocked in 5% dry milk in PBS with 0.1% Tween (PBST) for 1 h at room temperature and incubated at 4°C with primary antibody in PBST with 0.5% dry milk overnight. The primary antibodies used in this study were rabbit monoclonal RABMAB αPTBP1 (Abcam, Cambridge, UK), rabbit αSRSF3 (Assay Biotech, Sunnyvale, CA, USA), rabbit αGAPDH (Cell Signaling, Danvers, MA, USA), and rabbit αActin (Sigma‐Aldrich). Membranes were washed 3 × 10 min in PBST and incubated for 1 h at room temperature with a 1:10,000 dilution of secondary HRP‐conjugated αrabbit antibody (Pierce) in 0.5% dry milk in PBST. Membranes were washed 3 × 10 min in PBST and the proteins bands were visualized using SuperSignal West Dura Extended Duration Substrate (Thermo Fisher Scientific, Waltham, MA, USA) with Amersham Hyperfilm ECL (GE Healthcare). For SRSF3 band quantification, each band was firstly normalized to its respective Actin band and then related to its untreated control.

## 3 RESULTS

### 3.1 The tissue‐specific expression of PTBP1

**Figure 1:**
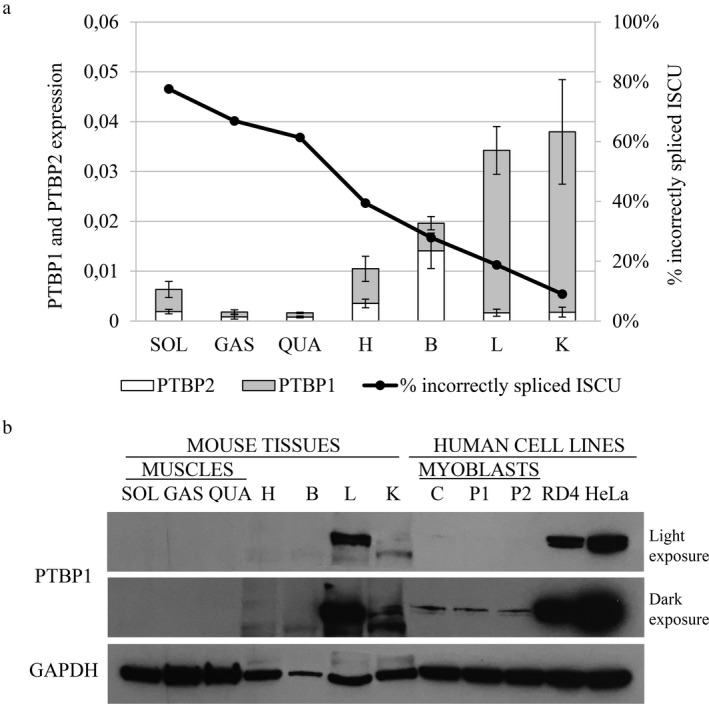
PTBP1 expression in mouse tissues. (a) PTBP1 and PTBP2 qRTPCR using cDNA from mouse tissues (*n* = 4–5 for each gene). The graph presents the average expression ±*SD* relative to the mouse GAPDH where each sample was run in triplicate. Percentage of incorrect splicing of *ISCU* data is from Rawcliffe et al. (2016). (b) PTBP1 Western blot using the nuclear extracts from the mouse tissues and protein samples from the healthy control (C) and HML patient (P1, P2) myoblasts, as well as RD4 and HeLa cells. To show the drastic difference in PTBP1 levels between cell lines both a light and dark exposure of the blot is shown. Tissues used were soleus (SOL), gastrocnemius (GAS), quadriceps (QUA), heart (H), brain (B), liver (L), and kidney (K). GAPDH was used as a loading control

Overexpression of *PTBP1* in RD4 cells has been shown to significantly diminish the mis‐splicing of an *ISCU* mini‐gene with the HML mutation. It was therefore suggested that PTBP1 can act as a repressor of the incorrect splicing of *ISCU* (Nordin et al., 2012). In a recent study, we showed that the levels of incorrectly spliced *ISCU* vary among a number of mouse tissues, where the highest level of incorrect splicing was observed in the slow fiber muscle soleus (Rawcliffe et al., 2016). In this study, we examined the same tissues for levels of *Ptbp1* RNA and protein to see whether there was a correlation between the levels of *ISCU* mis‐splicing and the levels of *Ptbp1* (Figure 1a,b). Since brain was among the examined tissues, we also analyzed the RNA levels of the brain‐specific *Ptbp1*‐paralog *Ptbp2*. When comparing the *Ptbp1* RNA levels to the levels of incorrectly spliced *ISCU*, we observed that a high expression of *Ptbp1* correlated with a low level of incorrectly spliced *ISCU* (Figure 1a). *Ptbp1* showed the lowest levels of RNA in the muscle, where we find up to 80% mis‐spliced *ISCU*, and the highest levels in the kidney and liver, where only 10%–20% of the transcripts are mis‐spliced (Figure 1a). A similar *Ptbp1* expression pattern was also observed on the protein level; however, the liver showed higher levels of PTBP1 than the kidney, while the remaining tissues showed no detectable levels of PTBP1 protein (Figure 1b). Human myoblasts showed higher levels of PTBP1 than the mouse muscle, while the muscle cancer cell line RD4 and the cervical cancer cell line HeLa showed significantly higher levels of PTBP1 (Figure 1b). The negative correlation of *Ptbp1* levels and *ISCU* mis‐splicing suggest that PTBP1 acts as a tissue‐specific splicing repressor of the incorrect splicing of *ISCU*.

### 3.2 PTBP1 binds with higher affinity to the mutated ISCU sequence compared to the wild type

**Figure 2:**
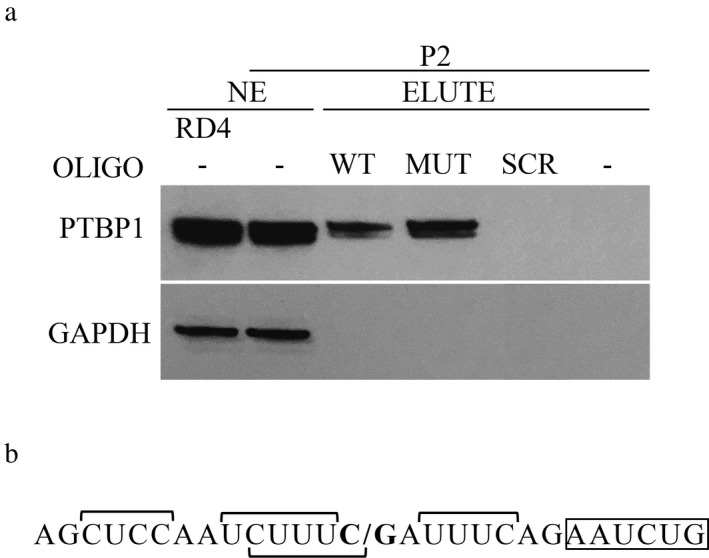
PTBP1 binding to normal and mutated *ISCU* RNA. (a) PTBP1 western blot of the eluted fractions (ELUTE) from the RNA pull‐down assay using nuclear extract (NE) from HML patient (P2) myoblasts and biotinylated RNA oligos; *ISCU* wild‐type oligo (WT), *ISCU* mutant oligo (MUT), and scrambled RNA oligo (SCR). A negative control with no oligo (−) is also included as well as NE from RD4 and P2. GAPDH was used as a loading control for NE and as a negative control for any nonspecific RNA–protein interactions. The pull‐down results were reproduced at least once for each oligo. (b) Sequence of the *ISCU* RNA oligos used in the pull‐down experiments with proposed PTBP1‐binding sites indicated by horizontal square brackets. **C/G** indicates the site of the HML mutation. The start of the HML pseudoexon is boxed

In a previous study, using RD4 cell nuclear extract, we showed that PTBP1 interacts with the wild‐type (WT) and mutant (MUT) *ISCU* sequence with a similar affinity (Nordin et al., 2012). In this study, we performed a pull‐down assay, using myoblast nuclear extract from HML patients. The pull‐down assay confirmed the interaction between PTBP1 and the WT and MUT *ISCU* sequence; however, we observed a stronger binding to the MUT sequence (Figure 2a). This is supported by the fact that the HML mutation, G > C, increases the number of pyrimidines in the polypyrimidine tract, generating a stronger PTBP1 binding motif (Figure 2b) (Garcia‐Blanco, Jamison, & Sharp, 1989; Keppetipola, Sharma, Li, & Black, 2012; Keppetipola et al., 2016; Patton, Mayer, Tempst, & Nadal‐Ginard, 1991). We observed significantly higher levels of PTBP1 in RD4 cells compared to the myoblasts (Figures 1b and 2a) which might indicate that the equal binding to the WT and MUT sequence using RD4 cells observed by Nordin et al. (2012) could be the result of saturated levels of PTBP1.

### 3.3 PTBP1 acts as a repressor of ISCU mis‐splicing

**Figure 3:**
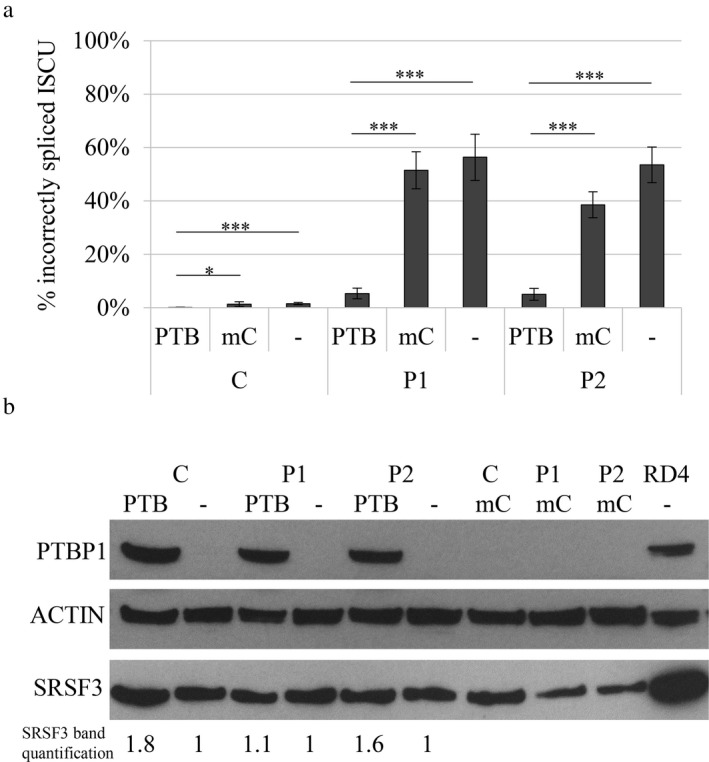
Incorrect splicing of *ISCU* in myoblasts overexpressing PTBP1. A lentivirus‐mediated expression vector PTBP1 (PTB) or a control expression vector mCherry (mC) was introduced into myoblasts from HML patients (P1, P2) and a healthy control (C). (a) Quantification of the incorrectly spliced *ISCU* by qRTPCR. The graph presents the mean percentage of incorrectly spliced *ISCU* ±*SD* from at least three independent experiments. (*p < 0.05, **p < 0.01, ***p < 0.001; Student's *t* test). (b) Western blot of PTBP1 overexpression in nontransduced and transduced myoblasts. ACTIN was used as a loading reference. Quantified levels of SRSF3 is indicated below the blot. Each band was normalized to ACTIN and related to its untreated control

**Figure 4:**
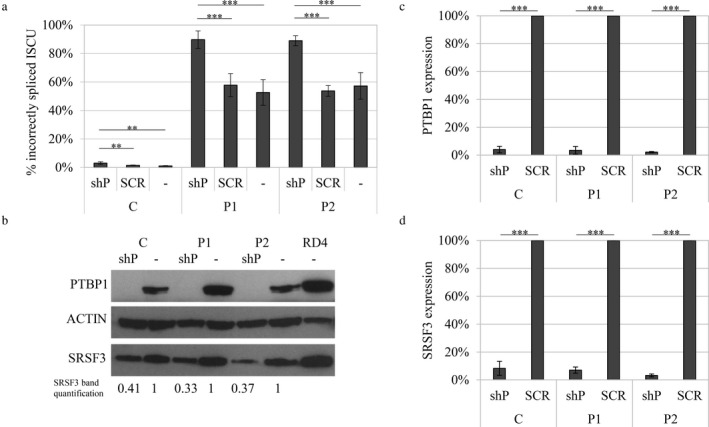
Incorrect splicing of *ISCU* in myoblasts with a knocked down PTBP1 expression. A lentivirus‐mediated expression vector for PTBP1 shRNA (shP) or scrambled shRNA (SCR) was introduced into myoblasts from HML patients (P1, P2) and a healthy control (C). (a) Quantification of incorrectly spliced *ISCU* by qRTPCR. The graph presents the mean percentage of incorrectly spliced *ISCU* ±*SD* from at least three independent experiments. (b) Western blot of PTBP1 knockdown in nontransduced and transduced myoblasts. ACTIN was used as a loading reference. Quantified levels of SRSF3 is indicated below the blot. Each band was normalized to ACTIN and related to its untreated control. (c) Relative PTBP1 RNA expression by qRTPCR (d) Relative SRSF3 RNA expression by qRTPCR (*p < 0.05, **p < 0.01, ***p < 0.001; Student's *t* test)

To investigate the PTBP1 repression of *ISCU* mis‐splicing in an endogenous context, we overexpressed as well as knocked down *PTBP1* in myoblasts from two HML patients and a healthy control using lentiviral transduction (Figures 3 and 4). The robust *PTBP1* overexpression decreased the levels of *ISCU* mis‐splicing in HML myoblasts eightfold, from approximately 40%–55% down to 5%, which is almost as low as in healthy control myoblasts (Figure 3a). The *PTBP1* overexpression also resulted in a small but variable, 1.1–1.8‐fold increase in SRSF3 protein (Figure 3b). We have previously shown that SRSF3 acts as an activator of *ISCU* mis‐splicing (Rawcliffe et al., 2016). However, the slight increase of SRSF3 will likely have a marginal effect on the level of mis‐splicing, considering the high levels of counteracting PTBP1 (Figure 3b). The overexpression of *PTBP1* also resulted in a significant eightfold decrease in *ISCU* mis‐splicing in the healthy control myoblasts, from approximately 1.5% down to 0.2% (Figure 3a).

Consistent with the result from the overexpression, knockdown of *PTBP1* resulted in a twofold increase in *ISCU* mis‐splicing to a total level of 90% in patient myoblasts as well as a threefold increase from approximately 1%–3% in the healthy control myoblasts (Figure 4a,b). In the myoblasts infected with shPTBP1 lentivirus, the *PTBP1* RNA levels were markedly decreased compared to the myoblasts infected with scrambled shRNA lentivirus (Figure 4c) or uninfected controls (data not shown). The knockdown of *PTBP1* also resulted in a drastic decrease in the *SRSF3* mRNA and a more moderate twofold decrease in the SRSF3 protein (Figure 4b,d). This could influence the mis‐splicing of *ISCU*; however, the net outcome is an increase in the *ISCU* mis‐splicing from approximately 60% up to 90% (Figure 4a).

## 4 DISCUSSION

Alternative splicing contributes to the complex protein diversity of eukaryotes, where approximately 95% of all human genes are alternatively spliced (Fu & Ares, 2014; Pan, Shai, Lee, Frey, & Blencowe, 2008; Wang et al., 2008). The interaction of splicing factors, specific for regulatory sequences in the pre‐mRNA, define the splice sites where spliceosome assembly takes place (Black et al., 2003). However, due to the unique collection of splicing factors in a particular tissue, the same splice site is recognized differently in different cell types, making it hard to decipher a general “splicing code” (Chen & Manley, 2009; Fu & Ares, 2014). Therefore, studies on specific mutational events, that drastically change the splicing of a certain gene in a tissue‐specific manner, can contribute a small piece to the overall puzzle.

We have previously shown that the high levels of incorrect splicing of *ISCU* in skeletal muscle, compared to other examined tissues, can explain the muscle‐specific phenotype seen in HML patients (Drugge et al., 1995; Larsson et al., 1964; Nordin et al., 2011; Rawcliffe et al., 2016). Tissue‐specific alternative splicing is often the outcome of an elaborate interplay between the activators and repressors of splicing. However, the main shift in the splicing outcome ratio often occurs when the repressor for a specific splicing event is removed, as opposed to the removal of the activator, making the repressor, in most cases, the dominant regulator of the splicing event (Coelho et al., 2015). PTBP1 and PTBP2 belong to the hnRNP protein family that are known to act as repressors of tissue‐specific alternative exons in a wide range of targets (Ling et al., 2016; Wagner & Garcia‐Blanco, 2001). PTBP1 binds to pyrimidine tracts, where longer pyrimidine tracts result in stronger binding (Chan & Black, 1997; Keppetipola et al., 2012, 2016; Markovtsov et al., 2000). PTBP1 is downregulated in muscle tissue and has been shown to repress the inclusion of muscle‐specific alternative exons (Boutz, Chawla, Stoilov, & Black, 2007; Lin & Tarn, 2011; Lustig et al., 2014; Patton et al., 1991; Reardon et al., 2011; Saulière, Sureau, Expert‐Bezançon, & Marie, 2006; Spellman, Llorian, & Smith, 2007; Spellman et al., 2005; Taniguchi et al., 2015).

To examine whether PTBP1 may act as the major repressor in the mis‐splicing of mutant *ISCU,* we compared the expression pattern of *PTBP1* to the levels of *ISCU* mis‐splicing in mouse tissues. We could indeed see a negative correlation between the levels of PTBP1 and *ISCU* mis‐splicing, supporting the idea that PTBP1 acts as a repressor of pseudoexon inclusion in HML. The skeletal muscles show the highest levels of mis‐splicing and the lowest levels of PTBP1, while the opposite was observed in the liver and kidney. The importance of PTBP1 in the regulation of endogenous *ISCU* mis‐splicing was further demonstrated by overexpression or knockdown of *PTBP1* in myoblasts from HML patients and a healthy control. Overexpression of *PTBP1* resulted in a drastic decrease in *ISCU* mis‐splicing in patient myoblasts. The opposite was seen when *PTBP1* was knocked down, with a twofold increase in *ISCU* mis‐splicing to a total level of 90% in patient myoblasts. Additionally, significant effects on levels of *ISCU* mis‐splicing was also observed in the control myoblasts. These results, together with the tissue‐specific expression of PTBP1, supports the hypothesis of PTBP1 as a dominant repressor of *ISCU* mis‐splicing.

We have previously suggested that the higher levels of SRSF3 in the soleus compared to the gastrocnemius and quadriceps could explain the higher levels of *ISCU* mis‐splicing in the soleus (Rawcliffe et al., 2016). As seen in a previous study, we also observed an inter‐regulatory connection between SRSF3 and PTBP1, where the knockdown of one results in decreased levels of the other (Guo, Jia, & Jia, 2015). Additionally, both SRSF3 and PTBP1 are known to self‐regulate their own expression by targeting the splicing of their own mRNA, further demonstrating the complexity of alternative splicing (Jumaa & Nielsen, 1997; Spellman et al., 2005; Wollerton, Gooding, Wagner, Garcia‐Blanco, & Smith, 2004).

As shown previously, incorrect splicing of *ISCU* does occur to a low degree in healthy control tissues (Mochel et al., 2008; Nordin et al., 2011). We observed similar low levels of *ISCU* mis‐splicing in control myoblasts, as well as a significant increase when *PTBP1* was knocked down. Most likely, the intronic cryptic splice sites give rise to leakage of *ISCU* transcript containing the pseudoexon, where the removal of PTBP1 splicing repression increases the leakage. However, to explain the drastic increase in mis‐splicing observed in patient cells, the mutation must not only activate the cryptic splice sites, but also alter the local splice site‐binding motif in favor of a splicing activator. The low levels of PTBP1 in the skeletal muscle, would then allow for the binding of a splicing activator(s), with a higher affinity for the mutant sequence, resulting in increased retention of the pseudoexon. Splicing activators that have been shown to compete with PTBP1 for binding of polypyrimidine tracts upstream of alternatively spliced exons could be potential candidates. Earlier pull‐down experiments revealed a number of RNA binding factors, aside from PTBP1, that interact with the wild‐type and mutant *ISCU* sequence, including MATR3, RBM39, and IGF2BP1 (Nordin et al., 2012). IGF2BP1 was the only one of these factors that showed a higher affinity for the mutant *ISCU* sequence but IGF2BP1 is not a known splicing factor (Nordin et al., 2012). Another interesting candidate is the U2 auxiliary factor (U2AF) complex, which is made up of the two subunits U2AF35 and U2AF65. U2AF65 has been shown to compete with PTBP1 for binding to polypyrimidine tracts at alternatively spliced exons and to promote splicing in the absence of PTBP1. (Pacheco et al., 2004; Saulière et al., 2006; Shao et al., 2014; Sharma, Falick, & Black, 2005). Other interesting candidates to explore include RBM4, RBM24, and MBNL1, which all have determinant roles in muscle‐specific splicing (Konieczny, Stepniak‐Konieczna, & Sobczak, 2014; Lin & Tarn, 2011; Yang et al., 2014).

Increased knowledge about how different splicing factors contribute to specific diseases may aside from increasing our knowledge of how the splicing machinery works, reveal new potential targets in the treatment of the diseases (Cieply & Carstens, 2015). Today, a common strategy to pharmacologically repress splicing defects is to use antisense oligonucleotides (ASOs). For HML, *ISCU*‐specific ASOs have been shown to decrease the levels of incorrect transcript in both fibroblasts and myotubes from HML patients, resulting in increased levels of ISCU protein (Holmes‐Hamptom et al., 2016; Kollberg et al., 2009; Sanaker, Toompuu, McClorey, & Bindoff, 2012). ASOs, along with identification of key splicing factors, represents essential contributions in the search for a treatment of HML as well as other diseases caused by splicing defects.

In summary, our results show that PTBP1 acts as a dominant repressor of *ISCU* mis‐splicing and that the high level of mis‐splicing in the skeletal muscle is most likely caused by the absence of PTBP1. As splicing is a highly intricate regulatory process, involving a plethora of splicing factors, the precise ratio of PTBP1 and other key factors, that are specific for each tissue, will determine the level of *ISCU* mis‐splicing. The effect of potential activators, in the regulation of *ISCU* mis‐splicing, needs to be further explored.

## CONFLICT OF INTEREST

The authors declare no conflicts of interest.
